# Geometric Analysis of Complex Endovascular Treatment of a Successfully Completed Residual Post-Type A Aortic Dissection

**DOI:** 10.1055/s-0042-1743201

**Published:** 2022-08-07

**Authors:** Alice Finotello, Bianca Pane, Mauro Di Bartolo, Rachele Del Pizzo, Simone Mambrini, Giovanni Pratesi, Giovanni Spinella

**Affiliations:** 1 Department of Surgical and Integrated Diagnostic Sciences, University of Genoa, Genoa, Italy; 2 Vascular and Endovascular Surgery Unit, Ospedale Policlinico San Martino, University of Genoa, Genoa, Italy

**Keywords:** residual Type A dissection, TEVAR, geometric analysis

## Abstract

We describe a case of complex multistep endovascular treatment of a post-Type A thoracoabdominal dissected aneurysm. Volume analysis documents true and false lumen improvements during follow-up. Centerline tortuosity of the aorta and of the iliac arteries straightens after endovascular treatment completion. In addition, analysis of stent-graft remodeling reveals the stent-graft tendency to spring back to its original status together with a caudal migration of the fenestrated body.

## Introduction

Endovascular treatment offers encouraging results in complex aortic pathologies such as thoracoabdominal aneurysms (TAAs) or aortic dissections (ADs). 1 In clinical practice, the results are evaluated in terms of intraprocedural technical success and 30-day mortality, long-term survival, and need for reinterventions. 2 However, the literature increasingly shows how close integration between geometric analysis and clinical indications can better assess treatment outcomes and, therefore, improve results in the near future. 3 4

We report a case of endovascular treatment of a post-Type A residual AD studied with geometric analysis during follow-up to obtain information on aortic remodeling.

## Case Presentation

**Fig. 1:**
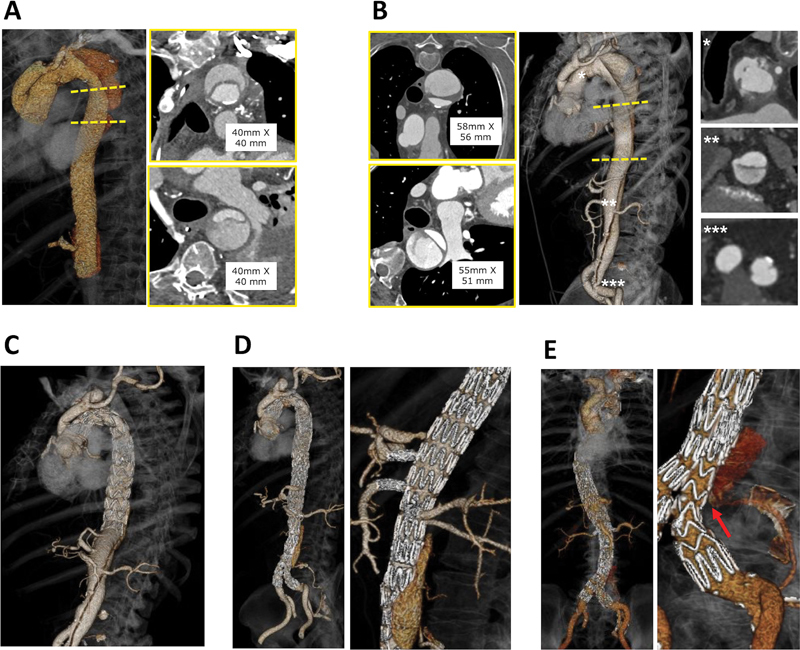
Comparison between CTA1 ( **A** ) and CTA2 ( **B** ) with increasing of the descending thoracic aortic diameter due to the presence of residual tears at level of aortic arch (*), right renal artery (**), and left common iliac artery (***). Three-dimensional rendering of CTA3 ( **C** ) and CTA5 ( **D** ) after thoracic endovascular aortic repair and fenestrated endovascular aortic repair. ( **E** ) CTA7 shows treatment success at medium-term follow-up. Red arrow highlights the hypertrophic remodeling of the collateral lumbar artery. CTA, computed tomography angiography.

A 75-year-old male previously treated with open surgery for a symptomatic Type A AD was admitted to our hospital for residual dissected TAA diagnosed by 1-month follow-up computed tomography angiography (CTA1), with increase in diameter from 40 × 40 mm to 58 × 56 mm at 6-month follow-up (CTA2). Imaging revealed residual dissection tears at the level of the aortic arch, right renal artery, and left common iliac artery ( Fig. 1A, B ).

A multistep hybrid aortic arch repair was planned, with left carotid-subclavian bypass and thoracic endovascular aortic repair plus proximal subclavian embolization with coils.

The patient received two Relay endoprostheses (Bolton Medical, Sunrise, FL) with proximal landing zone in Ishimaru zone 0 and distal landing zone 70 mm above the celiac trunk. No endoleaks were detected in postoperative CTA (CTA3, Fig. 1C ).

Six-month follow-up CTA (CTA4) was performed to schedule the second step treatment, with extension of the thoracic module with ZTA 34–30–161 endoprosthesis (Cook Medical, Bloomington, IN), a custom-made fenestrated stent-graft (Fen-Thoraco-Abdominal-Graft G38035; Cook Medical), a bifurcated aortic body (AAA-Body-Inverted-Leg G32596; Cook Medical), an iliac extension branch (ZSLE-20–74-ZT; Cook Medical), and four ADVANTA V12 (Atrium Medical Corporation) stent bridges.

Postprocedural CTA scan (CTA5) demonstrated technical success ( Fig. 1D ).

Follow-up CTAs were done at 6 (CTA6) and 16 (CTA7) months postoperatively ( Fig. 1E ). False lumen patency was detected, in corresponding to a lumbar artery which most likely fed the spinal cord, thus preventing spinal cord ischemia ( Fig. 1E ).

Geometrical analysis methodologies to analyze true lumen (TL) and false lumen (FL) and stent surfaces were derived according to previous work of our group. 4

TL centerline tortuosity, length of the whole centerline, and length of the centerline covered by the endograft(s) were automatically computed. Centerline tortuosity increased from 0.40 to 0.45 during the first 6-month follow-up after open surgery and continued to grow at CTA3 (0.46), before fenestrated endovascular aortic repair (FEVAR) (0.55), and immediately after (0.63). Following FEVAR, it straightened to 0.53 (CTA6) and to 0.51 (CTA7).

**Fig. 2:**
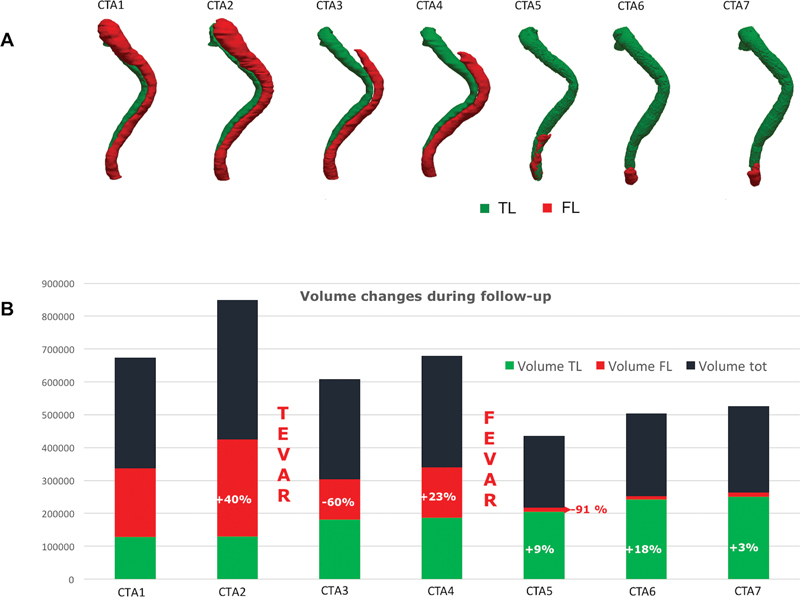
( **A** ) Three-dimensional surface reconstructions showing true lumen (TL) (green) and false lumen (FL) (red) for computed tomography angiographies (CTAs) 1–7. ( **B** ) Computation of volume of TL (green), FL (red), and total lumen (black). Percentages refer to volume changes computed in comparison with the previous CTA.

Volumes of TL and FL were automatically quantified as well, to assess how the treatments affected the aortic remodeling ( Fig. 2 ). CTA2 performed 6 months after open surgery revealed an increase in the FL of approximately 40% if compared with CTA1.

Conversely, immediately after the first-step endovascular treatment (CTA3), FL volume decreased by 60%, whereas a few months later during follow-up control (CTA4) it showed a +23% increase due to the presence of residual tears. After FEVAR (CTA5), FL volume reduction reached 91% if compared with the previous angiographic control (CTA4).

Conversely, TL remained almost stable from CTA1 to CTA2, whereas, immediately after first-step endovascular repair (CTA3) and after the FEVAR procedure, it experienced an increase. The same trend occurred during follow-up examinations with a +18% and +3% TL increase at CTA6 and CTA7, indicating a favorable remodeling of the TL over time. As a consequence of the TL expansion, total volume also increased ( Fig. 2B ).

**Fig. 3:**
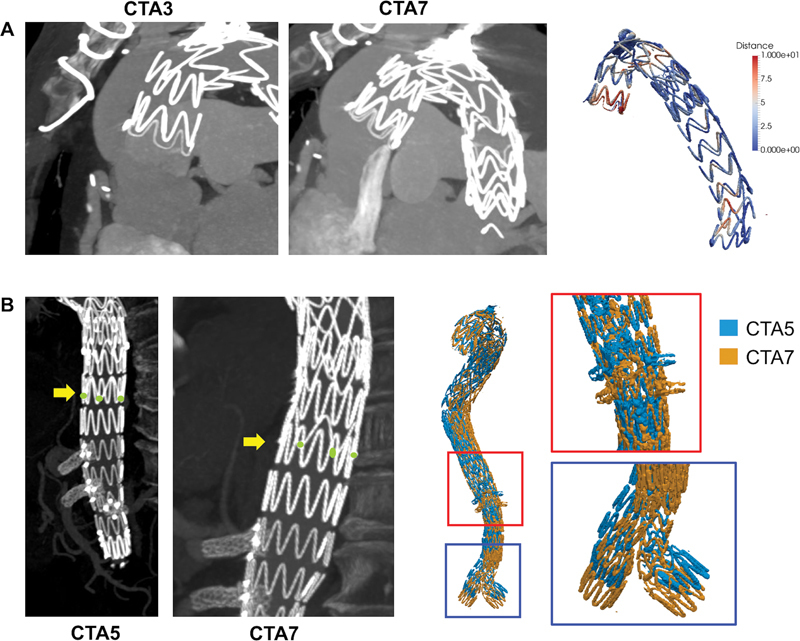
( **A** ) Pointwise distance computed between stent-graft surfaces extracted from CTA3 and CTA7 shows 10 mm difference at proximal level and ( **B** ) caudal migration of the fenestrated body between CTA5 and CTA7. CTA, computed tomography angiography.

Stent-graft surfaces that were extracted during the various follow-up steps and superimposed by rigid registration were compared by means of pointwise minimum distance algorithm. As shown in Fig. 3 , a progressive sliding of the stent proximal portion in its lateral direction was observed, from CTA3 to CTA4 (+5 mm) and to CTA7 (+10 mm). In addition, caudal migration of the fenestrated body from CTA5 to CTA7 (+9.2 mm) with a reduction of the overlap portion was observed. As a consequence, the angles between the fenestrated body and the stent bridges became more acute from CTA5 to CTA7 (celiac trunk: 14 degrees; superior mesenteric artery: 5 degrees; renal arteries: 4 degrees).

## Discussion

A clinical case of a hybrid aortic arch treatment and totally endovascular repair for a residual post-Type A dissected TAA has been described. Three main tears which supplied the FL were identified, with the largest located at the level of the aortic arch while the other two were found at the level of the right renal artery and of the left common iliac artery. We therefore planned a multistep endovascular treatment with the aim of closing all tears and therefore promoting FL thrombosis.

The geometric analysis of the volumes showed that at 7 months of follow-up after cardiac surgery, the volume of the FL, supplied by three secondary tears, increased by 40% compared with the first postoperative CTA performed after the ascending aorta replacement, reaching the maximum diameter of 70 mm in the descending thoracic aorta, with growth localized also at the level of the abdominal aorta, suggesting that the coverage of tears proximal to the level of the aortic arch is not always effective in promoting good aortic remodeling. 5

In fact, at 6 months of follow-up after the hybrid treatment, a new increase in the FL volume was observed due to the presence of secondary tears which fed the FL at the level of the abdominal aorta where the aortic collateral vessels originate.

Closure of the secondary tears can be performed by means of a totally endovascular treatment, even if extended coverage may expose patients to an increased risk of paraplegia. Multistep treatment, when feasible, 6 may reduce that risk, allowing stepwise accommodation by the body.

Moreover, the geometric analysis regarding stent-graft remodeling has highlighted the tendency of the stent-graft to remodel the physiological curvature of the aortic arch. This outcome is also confirmed by the reduction of centerline tortuosity along the entire aortic axis after FEVAR. In fact, stent-graft positioning in a highly curved artery (e.g., the aortic arch) may exert a force, especially at endograft landing zones, due to the inherent tendency of the stent-graft itself to recover its original straight configuration, possibly causing increase in stress to the vessel wall and ultimately leading to endograft-related vessel injuries. 7 Moreover, a caudal migration of the fenestrated component has also been observed, which should be read in view of the complications that can occur during follow-up, for example, Type III endoleaks or complications of the stent bridge fenestrations. 8 To date, we have not yet observed these complications, but during follow-up, if this trend is confirmed, reintervention may be necessary.
